# Extraction, Isolation, Structural Characterization and Anti-Tumor Properties of an Apigalacturonan-Rich Polysaccharide from the Sea Grass *Zostera caespitosa* Miki

**DOI:** 10.3390/md13063710

**Published:** 2015-06-11

**Authors:** Youjing Lv, Xindi Shan, Xia Zhao, Chao Cai, Xiaoliang Zhao, Yinzhi Lang, He Zhu, Guangli Yu

**Affiliations:** 1 Key Laboratory of Marine Drugs, Ministry of Education, School of Medicine and Pharmacy, Ocean University of China, Qingdao 266003, China; E-Mails: lvyoujing1988@163.com (Y.L.); shanxindi@hotmail.com (X.S.); zhaoxia@ouc.edu.cn (X.Z.); caic@ouc.edu.cn (C.C.); zhxl819@163.com (X.Z.); langyinzhi@163.com (Y.L.); elden0203@foxmail.com (H.Z.); 2 Shandong Provincial Key Laboratory of Glycoscience and Glycotechnology, Ocean University of China, Qingdao 266003, China

**Keywords:** *Zostera caespitosa* Miki, apigalacturonan, oligosaccharides, ESI-CID-MS^2^, anti-angiogenesis, immunoregulation

## Abstract

An apigalacturonan (AGA)-rich polysaccharide, ZCMP, was isolated from the sea grass *Zostera caespitosa* Miki. The depolymerized fragments derived from ZCMP were obtained by either acidic degradation or pectinase degradation, and their structures were characterized by electrospray ionization collision-induced-dissociation mass spectrometry (ESI-CID-MS^2^) and nuclear magnetic resonance (NMR) spectroscopy. The average molecular weight of ZCMP was 77.2 kD and it consisted of galacturonic acid (GalA), apiosefuranose (Api), galactose (Gal), rhamnose (Rha), arabinose (Ara), xylose (Xyl), and mannose (Man), at a molar ratio of 51.4꞉15.5꞉6.0꞉11.8꞉4.2꞉4.4꞉4.2. There were two regions of AGA (70%) and rhamnogalacturonan-I (RG-Ι, 30%) in ZCMP. AGA was composed of an α-1,4-d-galactopyranosyluronan backbone mainly substituted at the O-3 position by single Api residues. RG-Ι possessed a backbone of repeating disaccharide units of →4GalAα1,2Rhaα1→, with a few α-l-arabinose and β-d-galactose residues as side chains. The anti-angiogenesis assay showed that ZCMP inhibited the migratory activity of human umbilical vein endothelial cell (HUVECs), with no influence on endothelial cells growth. ZCMP also promoted macrophage phagocytosis. These findings of the present study demonstrated the potential anti-tumor activity of ZCMP through anti-angiogenic and immunoregulatory pathways.

## 1. Introduction

Angiogenesis plays a critical role in tumor growth and metastasis [1]. Previous reports have shown that several polysaccharides can inhibit angiogenesis via different signaling pathways [2,3,4,5]. Plant polysaccharides are ideal candidates as immunomodulators in anti-tumor therapy because of their macrophage modulatory effects and relative non-toxicity [6]. Alga-derived polysaccharides exhibit a wide range of bioactivities and it is feasible to find potential anti-tumor drugs from marine polysaccharides.

Apigalacturonan (AGA) is a kind of Apiose-rich pectin that exclusively occurs in a small number of aquatic monocots. Two types of AGA, namely, lemnan and zosterin, have been extracted from the duckweed, *Lemna minor* [7,8,9] and the marine phanerogam, *Zostera marina* [10,11,12], respectively. Both of them possess a backbone comprising α-1,4-d-galactopyranosyluronan. The structure of lemnan consists of a hairy region composed of β-1,3′-Api*_f_*, terminal and α-1,5-linked Ara*_f_*, terminal, β-1,3- and β-1,4-linked Gal*_p_*, terminal and β-1,4-linked Xyl*_p_* [7]. The structure of zostein has been extensively investigated in the 1960s and 1970s [11,12,13]; however, the specific linkage between side Ara*_f_* residues remained unknown until 2010, when Gloaguen reported that the side chains were composed of 1,2-linked Api*_f_* oligosaccharides [10].

Lemnan and zosterin exhibit a wide range of physiological activities. Lemnan imparts a positive effect on the immune system by activating the phagocytosis [8] and the inflammatory response [14]. On the other hand, zosterin strongly suppresses the proliferation, migration and invasion of A431 human epidermoid carcinoma cells by inhibiting the expression of metalloproteases [10]. Zosterin also possesses high metal-binding activity [15] and disrupts protein-synthesis in mouse liver cells [16].

*Zostera caespitosa* Miki (*Z. caespitosa* Miki) is a marine phanerogam and widely distributed in the coastal area of Liaoning, China, the southern coast of Japan, and the eastern coast of North Korea. It is one of most important species of *Zostera*; however, information on polysaccharides from *Z. caespitosa* Miki has not been reported. In the present study, an AGA-rich polysaccharide, ZCMP, was extracted and purified from *Z. caespitosa* Miki and its structure was determined. The anti-tumor activity of ZCMP was also evaluated by using anti-angiogenesis and macrophage phagocytosis assays.

## 2. Results and Discussion

### 2.1. Extraction, Purification and General Analysis of ZCMP

**Figure 1:**
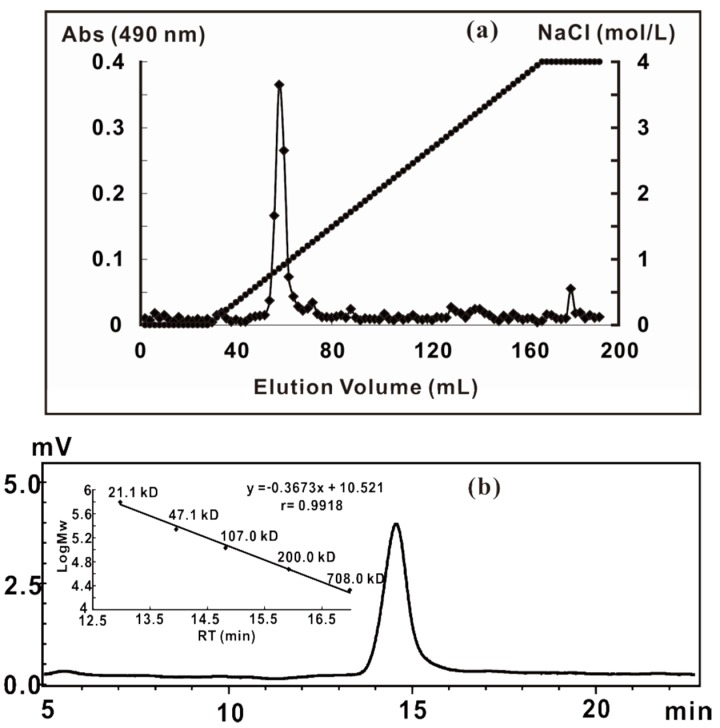
Separation and purification of ZCMP from *Z. caespitosa* Miki. (**a**) Elution profiles of ZCMP on a Q-Sepharose Fast Flow ion-exchange chromatography column; (**b**) The average molecular weight of ZCMP was determined using the High Performance Gel Permeation Chromatography (HPGPC) method on a Shodex OHpak SB804 column.

Ammonium oxalate is a calcium-chelating agent that is commonly used to increase pectin solubility. The yield of ZCMP extracted from *Z. caespitosa* Miki using 2% ammonium oxalate solution was 10.8% (w/w). ZCMP contained low levels of protein (4.3%) and sulfate (1.7%) and showed an average molecular weight of 77.2 kD. A single and symmetric peak on the Q-Sepharose Fast Flow (Figure 1a) and the Shodex OHpak SB-804 HQ column (Figure 1b) indicated that the extracted ZCMP was of high purity. Monosaccharide composition analysis demonstrated that ZCMP was composed of galacturonic acid (GalA), apiose (Api), galactose (Gal), rhamnose (Rha), arabinose (Ara), xylose (Xyl) and mannose (Man) at a molar ratio of 51.4꞉15.5꞉6.0꞉11.8꞉4.3꞉4.4꞉4.2 (Table 1), which was similar to that of lemnan and zosterin [7,10].

### 2.2. Preparation of ZCMP-Derived Oligosaccharides

#### 2.2.1. Degradation of ZCMP

**Figure 2:**
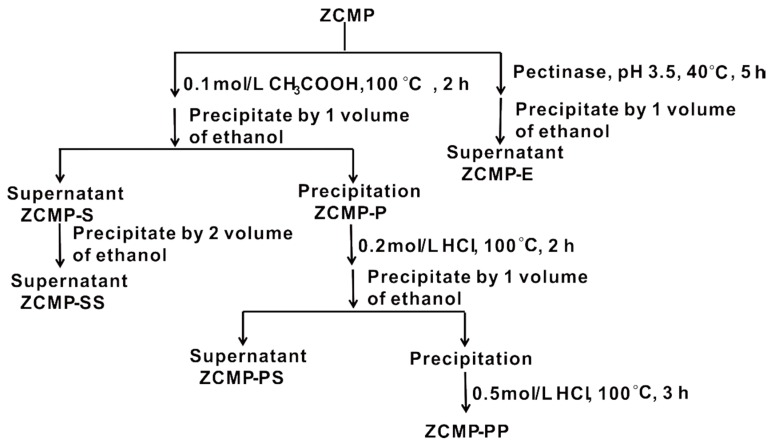
Flow chart of the degradation process of ZCMP.

ZCMP was shown to be sensitive under acidic conditions such as 0.1 mol/L HCl and H_2_SO_4_, and the Api residues were rapidly released as monosaccharides in our model experiment. A three-level acid solution (0.1 mol/L CH_3_COOH, 0.2 mol/L HCl, and 0.5 mol/L HCl) was added to degrade the polysaccharide progressively, and pectinase was also used to generate oligosaccharides with methyl esters and acetyl groups. The depolymerized oligosaccharides were collected by precipitation using different concentrations of ethanol. The degradation process of ZCMP is shown in Figure 2.

Monosaccharide composition analysis (Table 1) showed that the fractions ZCMP-P, ZCMP-PS, and ZCMP-SS contained different molar ratios of monosaccharides, indicating that they were derived from different regions of ZCMP. Api was the major monosaccharides released in the first step of hydrolysis, and only little Api in ZCMP-PS and ZCMP-P was detected, thus promoting us to speculate that the Api residues were at the terminal position or side chains. Meanwhile, Gal, Xyl, and Ara were mainly detected in ZCMP-SS and ZCMP-PS, which suggested that the three residues existed in the branches. ZCMP-PS mainly contained GalA and Rha with 7%–10% Gal and Ara residues, suggesting that the major component of ZCMP-PS was the fragment of RG-Ι. The content of GalA increased with the enhancement of acid strength and it was almost the only monosaccharide in ZCMP-PP, which indicated that GalA was present in the backbone of ZCMP structure.

**Table 1:** Molecular weight and monosaccharide analysis of ZCMP and its oligosaccharides.

	Molecular Weight (kD)	Monosaccharides (%)							
		Man	Rha	GlcA	GalA	Api	Gal	Xyl	Ara
ZCMP	77.2	4.2	11.8	-	51.4	15.5	6.0	4.4	4.3
ZCMP-SS	-	2.3	6.7	3.9	5.7	52.1	10.7	5.8	12.2
ZCMP-PS	-	3.0	27.0	5.0	39.9	2.5	10.1	4.7	7.8
ZCMP-P	23.4	3.9	5.8	0.9	79.9	2.9	3.1	1.2	2.2
ZCMP-PP	-	3.4	3.0	0.4	93.2	-	-	-	-

#### 2.2.2. Purification of ZCMP-Derived Oligosaccharides

**Figure 3:**
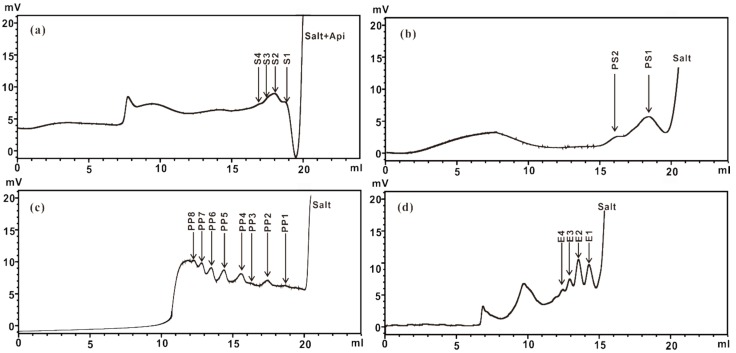
Low pressure gel-permeation chromatography of ZCMP-derived oligosaccharides from *Z. caespitosa* Miki. (**a**) ZCMP-S; (**b**) ZCMP-PS; (**c**) ZCMP-PP; (**d**) ZCMP-E.

The mixtures of ZCMP-derived oligosaccharides were fractionated by gel filtration chromatography (Figure 3). The proposed structural composition (abundance >10% in the ESI-MS spectrum) of oligosaccharides is presented in Table 2, which was based on the monosaccharide composition and ESI-MS analysis in the negative mode. Oligosaccharides with similar molecular mass and charge coexisted as one broad peak during gel-permeation chromatography, which was mainly due to the heterogeneous structure of ZCMP. Most of the Api coexisted in the salt peak and only minor Api-oligosaccharides were detected in ZCMP-S2-4 with a low polymerization degree (<5; Figure 3a).

**Table 2:** Components (abundance of >10% in the ESI-MS spectrum) of the oligosaccharide fractions degraded from ZCMP.

Fraction	Ions	Mw (H Form)	Dp	Composition
E1	272.05 (z = 2)	546.10	3	GalA_3_
	338.07 (z = 2)	678.14	4	GalA_3_Api
E2	448.08 (z = 2)	898.16	5	GalA_5_
	360.06 (z = 2)	722.12	4	GalA_4_
	342.40 (z = 3)	1030.10	6	GalA_5_Api
	386.41 (z = 3)	1162.23	7	GalA_5_Api_2_
E3	536.09 (z = 2)	1074.18	6	GalA_6_
	401.07 (z = 3)	1206.21	7	GalA_6_Api
	445.09 (z = 3)	1338.27	8	GalA_6_Api_2_
E4	415.74 (z = 3)	1250.22	7	GalA_7_
	355.56 (z = 4)	1426.24	8	GalA_8_
	459.75 (z = 3)	1382.25	8	GalA_7_Api
	503.76 (z = 3)	1514.28	9	GalA_7_Api_2_
	547.78 (z = 3)	1646.34	10	GalA_7_Api_3_
	591.79 (z = 3)	1778.37	11	GalA_7_Api_4_
S1	149.05 (z = 1)	150.05	1	Api
S2	281.10 (z = 1)	282.10	2	Api_2_
S3	413.14 (z = 1)	414.14	3	Api_3_
S4	545.18 (z = 1)	546.18	4	Api_4_
PS1	339.09 (z = 1)	340.09	2	GalARha
	369.10 (z = 1)	370.10	2	GalA_2_
	545.10 (z = 1)	546.10	3	GalA_3_
PS2	661.17 (z = 1)	662.17	4	GalA_2_Rha_2_
	721.12 (z = 1)	722.12	4	GalA_4_
PP1	193.08 (z = 1)	194.08	1	GalA
PP2	369.10 (z = 1)	370.10	2	GalA_2_
	339.09 (z = 1)	340.09	2	GalARha
PP3	545.10 (z = 1)	546.10	3	GalA_3_
PP4	721.12 (z = 1)	722.12	4	GalA_4_
PP5	448.08 (z = 2)	898.16	5	GalA_5_
PP6	536.09 (z = 2)	1074.18	6	GalA_6_
PP7	624.10 (z = 2)	1250.20	7	GalA_7_
PP8	474.41 (z = 3)	1426.24	8	GalA_8_

### 2.3. ESI-CID-MS^2^ Analysis of the Oligosaccharides Derived from ZCMP

Several reports have summarized the major contributions of mass spectrometry to the structural elucidation of carbohydrates [17,18,19]. The formation of ^0,2^X and ^0,2^A ions requires hydrogen on C3-OH and occurs at the 4-linked monosaccharide residue [20,21]. ^1,3^A-type cleavage usually arises with 2-linked residues [22,23]. Reduction of the hemiacetal to alditol is a common method to determine the reducing terminal of oligosaccharides. A reducing terminal ion will have a 2 Da increment after reduction by sodium borohydride [24]. Therefore, the multistage mass spectrum facilitates in determining the linkages and sequences of oligosaccharides.

#### 2.3.1. ESI-CID-MS^2^ Analysis of the Sequences of Oligosaccharides from ZCMP-S

**Figure 4:**
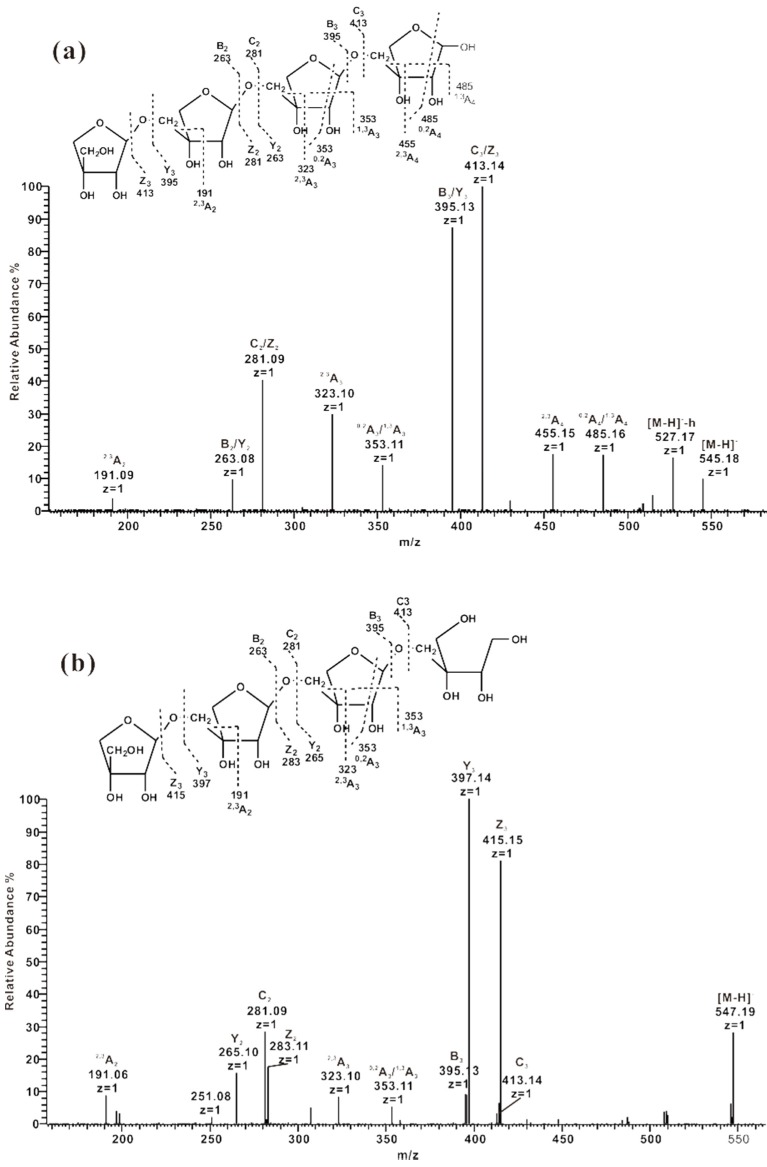
Negative-ion ESI-CID-MS^2^ product-ion spectra of the tetrasaccharide Api_4_ from ZCMP-S. (**a**) Sequence analysis of the tetrasaccharide Api_4_ at *m*/*z* 545; (**b**) Sequence analysis of the tetrasaccharide alditol at *m*/*z* 547.

A series of Api-oligosaccharides was released from ZCMP after CH_3_COOH hydrolysis and the ESI-CID-MS^2^ spectra of di-, tri- and hexa-saccharides were detected. The results demonstrated that all of them possessed the same fragment ion pattern. Taking the product-ion spectrum of Api_4_ (*m*/*z* 545) as an example (Figure 4), a series of ions of glycosidic bond cleavage at *m*/*z* 263 (B_2_/Y_2_), 281 (C_2_/Z_2_), 395 (B_3_/Y_3_) and 413 (C_3_/Z_3_) indicated a linear chain. In addition, a series of notably ^2,3^A type ions (*m*/*z* 191, 323, 455) were generated by cross-cleavage of the C2-C3 and C3-C4 bonds of Api, which in turn led to the loss of C_3_H_6_O_3_ of *m*/*z* 90. The ion at *m*/*z* 485 was deduced as ^1,3^A_4_ or ^0,2^A_4_ cleavage. Similarly, the ion at *m*/*z* 353 was deduced as ^1,3^A_3_ or ^0,2^A_3_ cleavage. Guo *et al.* [25] also determined a series of linear oligo-galatofuranoses by negative-ion ESI-CID-MS^2^. Cross-ring fragment ions of ^3,4^A and ^0,3^A-type fragment ions were observed as well and used in the identification of linkages between the β-D-(1→5)-linked Gal*_f_* oligosaccharides. Based on the proposed similar ion pattern of fragments, the linkage between Api residues was deduced to be 3′-linked. After reduction (Figure 4b), four glycosidic ions of tetrasaccharide Api_4_ shifted to *m*/*z* 415 (Z_3_), 397 (Y_3_), 283 (Z_2_) and 265 (Y_2_) from *m*/*z* 413, 395, 281 and 263, respectively. No cross-ring cleavage ions shifted after reduction, suggesting that all of these were produced from the non-reducing end.

The structures of lemnan and zosterin are restricted to algal species. Lemnan has a side chain of 3′-linked Api residues [8], whereas zosterin has a side chain of 2-linked Api residues [10]. However, the AGA obtained from *Z. caespitosa* Miki showed a similar side chain as that observed in lemnan.

#### 2.3.2. ESI-CID-MS^2^ Analysis of Oligosaccharides from ZCMP-PS

Even-numbered oligosaccharides with equal amounts of Rha and GalA were identified in the fractions of ZCMP-PS as GalA-Rha and GalA_2_Rha_2_, which indicated the presence of a repeating disaccharide unit. Its product ion spectra were acquired by ESI-CID-MS^2^.

**Figure 5:**
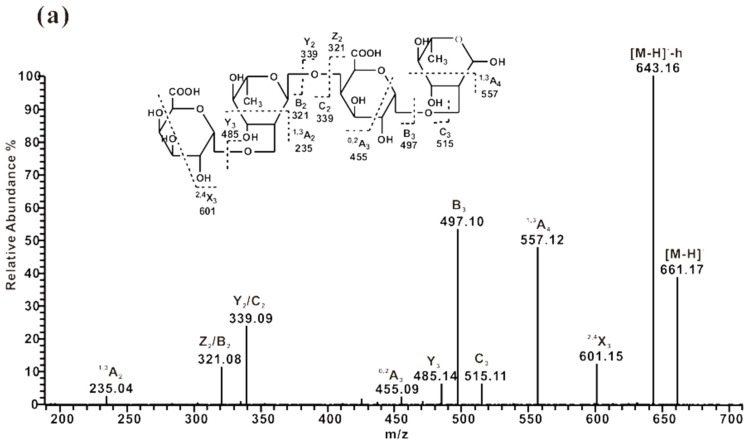

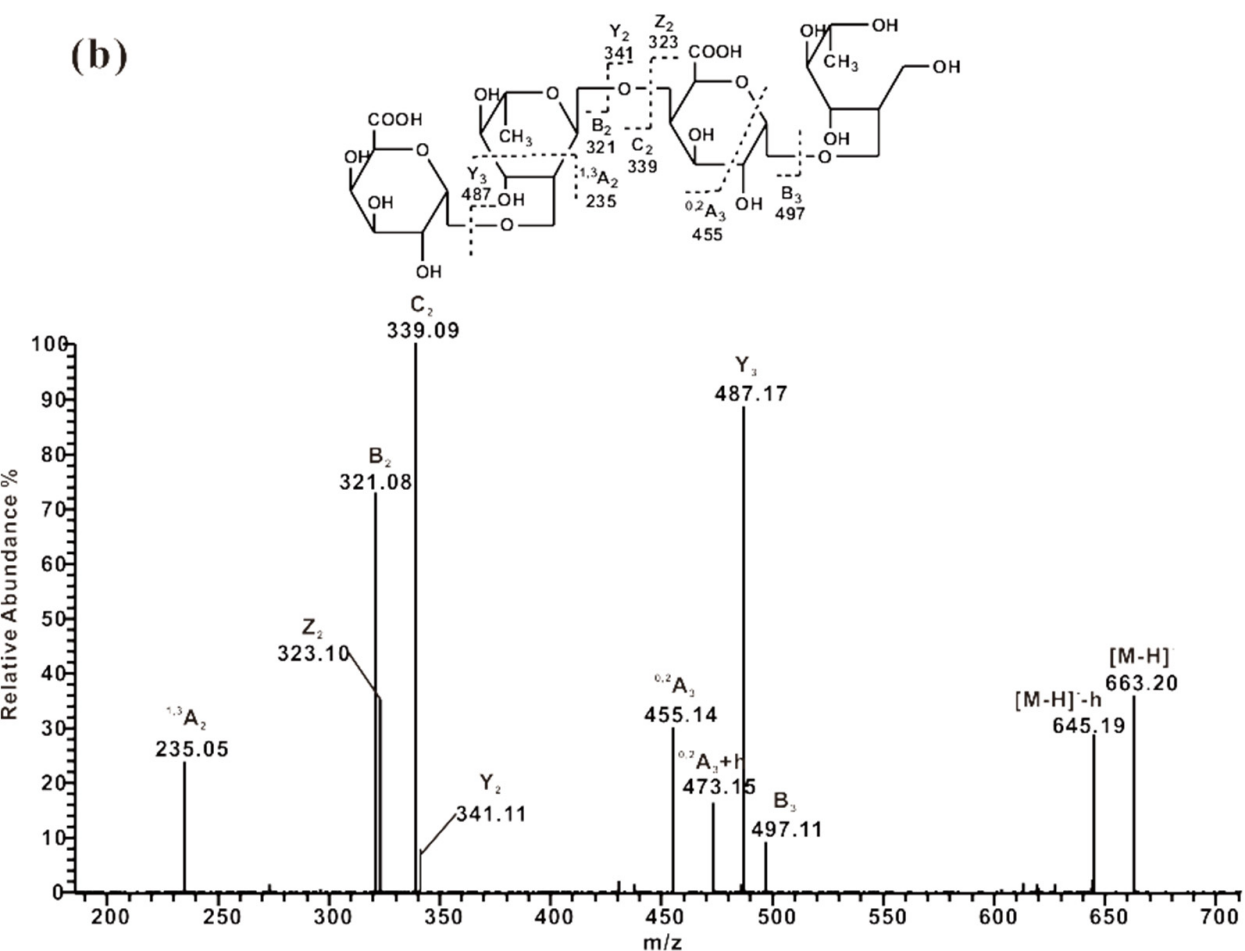
Negative-ion ESI-CID-MS^2^ product-ion spectra of the tetrasaccharide (GalA-Rha)_2_from ZCMP-PS. (**a**) Sequence analysis of (GalA-Rha)_2_ at *m/z* 661; (**b**) Sequence analysis of tetrasaccharide alditol at *m*/*z* 663.

The ESI-CID-MS^2^ spectrum (Figure 5) of the tetrasaccharide GalA_2_Rha_2_ (*m*/*z* 661) showed a series of ions of glycosidic bond cleavage at *m*/*z* 321 (B_2_/Z_2_), 339 (C_2_/Y_2_), 485 (Z_3_), 497 (B_3_), and 515 (C_3_), indicating a linear chain with repeating linkages of GalA and Rha. Comparison of the spectra of GalA_2_Rha_2_ (*m*/*z* 661) with its alditol (*m*/*z* 663) after reduction showed that the two glycosidic ions shifted to *m*/*z* 323 (Z_2_) and 487 (Y_3_) from *m*/*z* 321 and 485, respectively, thus revealing that Rha was at the reducing terminal. The ^1,3^A_4_ ion (*m*/*z* 557) from the reduced Rha and the ^1,3^A_2_ ion (*m*/*z* 235) from the internal Rha revealed the presence of 2-linked Rha. The ^0,2^A_3_ (*m*/*z* 455) and ^2,4^X_3_ (*m*/*z* 601) ions were the characteristic evidence for 4-linked GalA. A similar fragment ion pattern was observed in the ESI-CID-MS^2^ spectrum of the disaccharide GalA-Rha (Supplementary Figure 1). In the present study, GalA and Rha residues in the backbone of pectin were determined to be in the α-configurations, which was similar to the findings of previous NMR results [7,8,10,26,27]. Therefore, the structure of the main oligosaccharides in ZCMP-PS were identified as -[4)-α-GalA-(1→2)-α-Rha-(1]*_n_*-, which was assigned as the backbone of RG-Ι and was in agreement with the findings of previous reports [26,27].

#### 2.3.3. ESI-CID-MS^2^ Analysis of Oligosaccharides from ZCMP-PP

**Figure 6:**
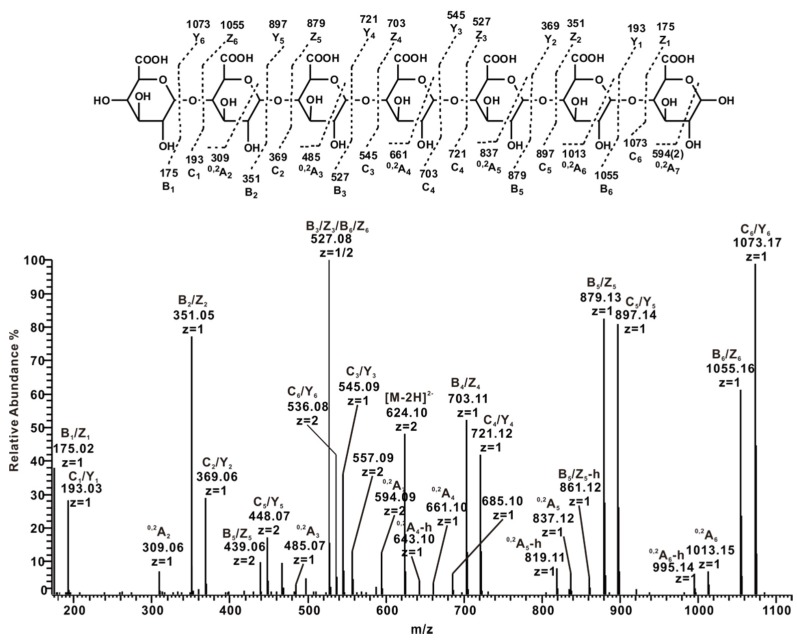
Negative-ion ESI-CID-MS^2^ product-ion spectrum of the heptasaccharide GalA_7_ derived from ZCMP-PP.

The backbone of ZCMP was completely degraded into the oligosaccharides ZCMP-PP by 0.5 mol/L HCl after removing the branches and the RG-Ι region. ZCMP-PP comprised a series of GalA oligosaccharides, except for a few GalA-Rha disaccharides (Table 2). The ESI-CID-MS^2^ spectra were obtained from disaccharides to octasaccharides, and all of these showed a similar fragment ions pattern. For instance, in the negative-ion production-ion spectrum of GalA_7_ at *m*/*z* 624.10 (Figure 6), a linear sequence was deduced from the major fragment ions *m*/*z* 175/193, 351/369, 527/545, 703/721, 879/897 and 1055/1073, which all had arisen from glycosidic bond cleavages. As described in previous reports, the formation of ^0,2^X and ^0,2^A ions requires hydrogen on C3-OH and only occurs at the 4-linked monosaccharide residue [17,28]. The observation of continuous cross-ring cleavage of ^0,2^A ions suggests that GalA oligomers in ZCMP-PP were homogenous 4-linked. All ^0,2^A ions were accompanied by ions derived from dehydration, e.g., ^0,2^A_3_, *m*/*z* 485/467 (weak); ^0,2^A_4_, *m*/*z* 661/643; ^0,2^A_5_, *m*/*z* 837/819; ^0,2^A_6_, *m*/*z* 1013/995; and ^0,2^A_7_, *m*/*z* 594/585 (double charged).

#### 2.3.4. ESI-CID-MS^2^ Analysis of Oligosaccharides from ZCMP-E

**Figure 7:**
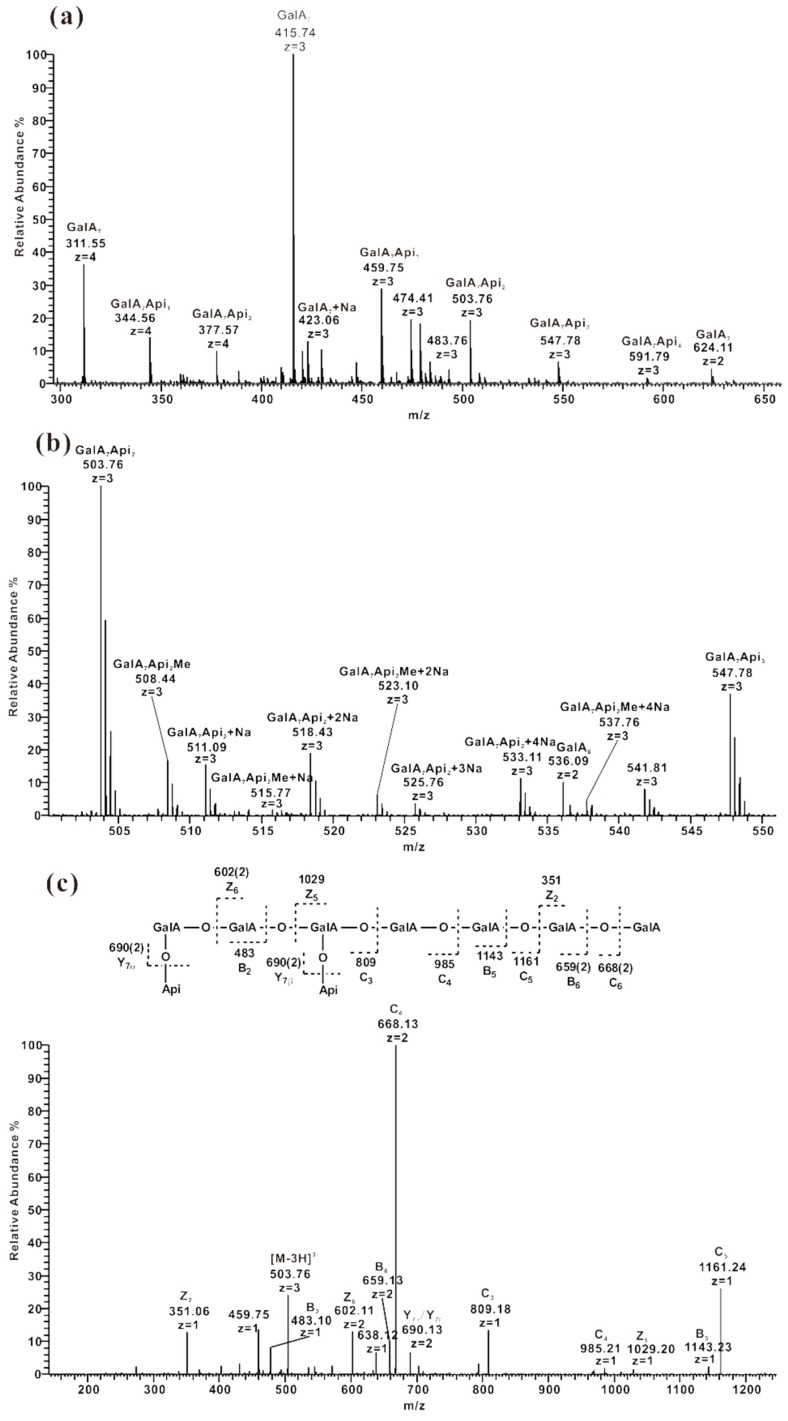
Negative-ion ESI-MS product-ion spectra of fractions from ZCMP-E. (**a**) Negative-ion ESI-MS spectrum of ZCMP-E4; (**b**) Five-fold magnification of the region between 500 and 550 *m*/*z* in the negative-ion ESI-MS spectrum of ZCMP-E; (**c**) Sequence analysis of nonasaccharide GalA_7_Api_2_ at *m*/*z* 503.76 (triple charged).

Pectinase can specifically cleave the glycosidic bond between GalA residues. ESI-MS analysis of pectinase hydrolysate ZCMP-E1-4 showed that all fractions were mixtures of different oligosaccharides. For example, GalA_7_, GalA_7_Api_1_, GalA_7_Api_2_, GalA_7_Api_3,_ and GalA_7_Api_4_ were observed in ZCMP-E4 (Figure 7a). The GalA residues are usually methyl esterified or acetylated partially at the O-2 and/or O-3 positions in pectin [29,30]. Weak fragment ions at *m*/*z* 508.44, 515.77, 523.10 and 537.76 (triple charged), assigned to [M − 3H]^3^^−^, [M − 4H + Na]^3^^−^, [M − 5H + 2Na]^3^^−^ and [M − 7H + 4Na]^3^^−^ of GalA_7_Api_2_Me, respectively, were found after magnifying the spectrum by five-fold (Figure 7b). Low abundance (<4%) of these peaks suggested that a small number of GalA residues were methyl esterified. No acetylated oligosaccharides were detected in E4.

To investigate the linkages between GalA residues and Api residues, ion at 503.76 (GalA_7_Api_2_, triple charged) from E4 was selected as precursor ion to get an ESI-CID-MS^2^ spectrum (Figure 7c). Ion at *m*/*z* 690 (double charged, Y_7α_/Y_7β_) confirmed that the Api residues were on the side chains. Ion detected at *m*/*z* 809 (C_3_) was assigned as GalA_3_Api_2_, indicating that there were four unsubstituted GalA residues on the terminal and the two Api residues were distributed on the other three GalA residues. The appearance of Z_6_ at *m*/*z* 602 (double charged) demonstrated that the disaccharide Api-GalA was on the terminal and the B_2_ ion at *m*/*z* 483 indicated that the trisaccharide Api-GalA-GalA was on the terminal. The deduced sequence of this nonasaccharide is shown in Figure 7c. The Api residues were randomly distributed on different GalA residues in the form of monosaccharides rather than oligosaccharides, indicating that the level of the Api oligosaccharides in ZCMP was relatively low, and most of Api residues were monosaccharides. The linkage between GalA and Api residues was not deduced due to the absence of cross-ring cleavages.

### 2.4. Methylation Analysis of ZCMP

The linkage between GalA and Api residues was also confirmed by carboxyl-reduction and methylation analysis (Table 3), in which 1,3,4,5-Ac_4_-2,6-Me_2_-d-galactitol_red_ was detected, suggesting that GalA residues were substituted at the O-3 position by Api. Large amounts of 1,4-Ac_2_-2,3,3′-Me_3_-Api*_f_* and lower amounts of 1,3′,4-Ac_3_-2,3-Me_2_-Api*_f_* were detected, indicating that there was a high level of terminal Api and a low level of 3′-linked Api residues in ZCMP. In addition, 1,4,5-Ac_3_-2,3-Me_2_-d-arabinitol, 1,4,5-Ac_3_-2,3-Me_2_-d-xylitol, 1,2,5-Ac_3_-6-deoxy-3,4-Me_2_-d-mannitol, 1,4,5-Ac_3_-2,3,6-Me_3_-d-galactitol, and 1,3,4,5-Ac_4_-2-Me_2_-d-arabinitol were detected, which originated from the 5-substituted Ara*_f_*, 4-substituted Xyl, 2-substituted Rha, 4-substituted Gal, and 3,5-substituted Ara residues, respectively.

**Table 3:** Methylation analysis of ZCMP.

Permethylated Alditol Acetate	Primary Mass Fragments (*m*/*z*)	Linkages	Molar Ratio
2,3,3′-Me_3_-Api *_f_*	118, 132, 161	Api *_f_*-(1→	12.81
2,3-Me_2_-Ara *_f_*	118, 129, 189	→5)-Ara *_f_*-(1→	14.26
2,3-Me_2_-Xyl		→4)-Xyl-(1→	
3,4-Me_2_-Rha	131, 190, 234, 304	→2)-Rha-(1→	17.75
2,3,6-Me_3_-Gal	118, 131, 173, 233	→4)-Gal-(1→	6.55
2,3,6-Me_3_-Gal_red_	118, 174, 234	→4)-GalA-(1→	32.40
2,6-Me_2_-Gal_red_	118, 130, 186, 306	→3,4)-GalA-(1→	8.35
2-Me-Ara *_f_*	118, 159, 201, 261	→3,5)-Ara *_f_*-(1→	3.07
2,3-Me_2_-Api *_f_*	118, 129, 189, 234	→3′)-Api *_f_*-(1→	4.82

Gal_red_: Gal residues generated from GalA residues by reduction with NaBD_4_.

### 2.5. NMR Analysis of ZCMP-SS

**Figure 8:**
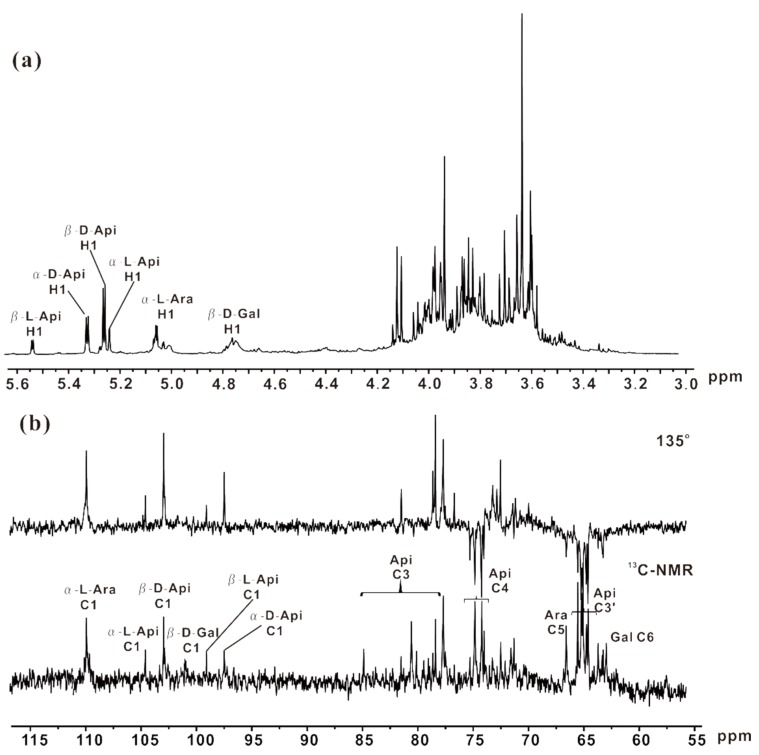
1D NMR spectra of ZCMP-SS. Spectral analysis was performed at 25 °C on a JEOL ECP 600 MHz spectrometer using acetone as internal standard. (**a**) ^1^H NMR spectrum and (**b**) ^13^C NMR and DEPT spectra.

**Figure 9:**
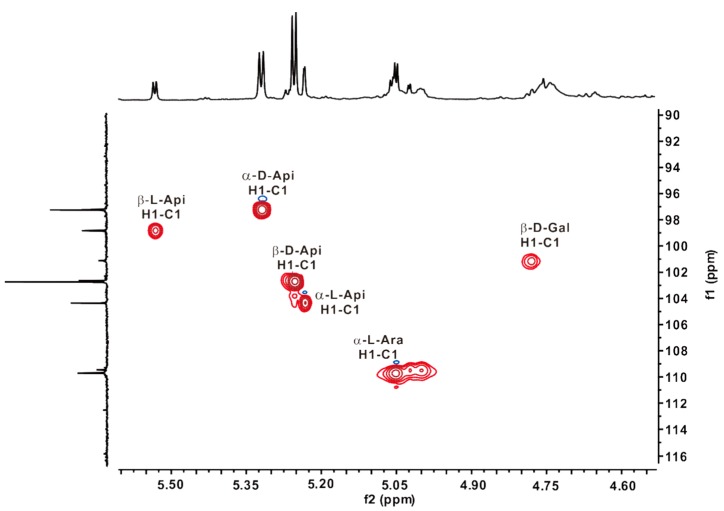
The ^1^H-^13^C HSQC spectrum of ZCMP-SS. Spectral analysis was performed at 25 °C on a JEOL ECP 600 MHz spectrometer using acetone as internal standard.

The structural features of ZCMP-SS were also characterized by using a combination of one-dimensional ^1^H NMR, ^13^C NMR, and Distortionless Enhancement by Polarization Transfer (DEPT) experiments (Figure 8), as well as heteronuclear two-dimensional ^1^H-^13^C Heteronuclear Multiple Quantum Coherence (HMQC) experiment. The proton-carbon correlation was assigned based on the HMQC spectrum (Figure 9), and seven cross peaks corresponding to the anomeric signals were clearly detected. Correlations between H1 at 5.24 ppm and C1 at 104.70 ppm, H1 at 5.33 ppm and C1 at 97.60 ppm, H1 at 5.54 ppm and C1 at 99.18 ppm, and H1 at 5.27 ppm and C1 at 103.08 ppm were assigned to α-l-Api*_f_*, α-d-Api*_f_*, β-l-Api*_f_*, and β-d-Api*_f_* respectively [10,31]. Its molar ratio was determined to be 1.0:3.0:4.0:1.3, based on the integral area ratio detected in ^1^H-NMR. No correlations of Api oligosaccharides in the side chains were detected due to its instability under acidic conditions. The anomeric proton signals of linked α-l-Ara and terminal α-l-Ara residues at 5.05 ppm and 5.02 ppm were correlated to the anomeric carbon signals at 110.1 ppm and 109.80 ppm, respectively. The correlation of H1 at 4.76 ppm with C1 at 101.44 ppm was assigned to β-d-Gal residues.

**Figure 10:**
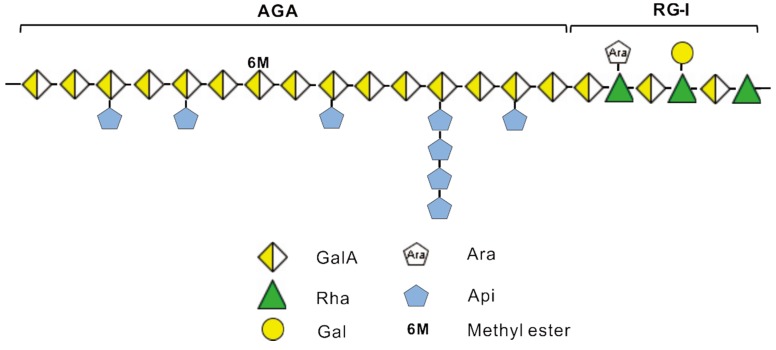
Proposed structural model of ZCMP.

According to the proposed general structural model for lemnan [7], zosterin [10], pectin [32], and the results obtained in the present study, we propose the following structural model for *Z. caespitosa* Miki polysaccharide ZCMP (Figure 10). ZCMP is composed of AGA and RG-Ι regions. AGA has a backbone of α-1,4-d-galactopyranosyluronan with an extremely low degree of etherification, whereas the side chains were linked to the O-3 of GalA by most of the single Api residues and minor short (1→3′)-linked β-d-Api oligosaccharides with different degree of polymerization (<5). RG-Ι has a backbone of repeating 4-linked GalA and 2-linked Rha with minor 5-linked α-l-Ara residues and 4-linked β-d-Gal residues as side chains.

### 2.6. ZCMP Inhibited the Migration of HUVECs

**Figure 11:**
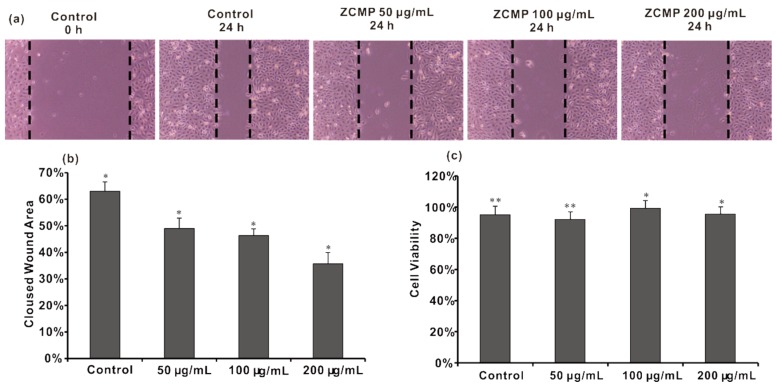
ZCMP inhibited the migration of HUVECs (**a**) HUVEC monolayer was scraped to generate a wound (0 h), and the cells were incubated with different concentrations of ZCMP (50 μg/mL, 100 μg/mL, 200 μg/mL) or vehicle (Control). After 24 h, the cells were imaged at 40× magnification. The wound areas at 0 and 24 h are indicated by dotted lines; (**b**) Quantification of the effect of ZCMP on HUVEC migration in the wound healing assay; (**c**) HUVEC viability was determined by using the MTT assay after incubating with different concentrations of ZCMP (50 μg/mL, 100 μg/mL, 200 μg/mL) or vehicle (Control). Each experiment was performed at least 3 times, and the values represent the mean ± S.D. * *P* < 0.05; ** *P* < 0.01, as determined by unpaired student’s *t*-test.

To assess the anti-angiogenic properties of ZCMP *in vitro*, its inhibitory effects on the chemotactic motility of human umbilical vein endothelial cell (HUVECs) were investigated using the wound-healing migration assay. As shown in Figure 11a, untreated HUVECs migrated into the wounded area of the cell monolayer, whereas ZCMP treatment significantly inhibited the HUVEC migration in a dose-dependent manner (Figure 11a,b).

HUVEC viability was tested to determine whether the migration inhibitory effect was the result of the inhibition of HUVEC proliferation after treatment with various concentrations of ZCMP for 24 h. As shown in Figure 11c, ZCMP had no significant effect on the viability of HUVECs.

Angiogenesis plays an important role in providing nutrients and oxygen to the growing tumor, whereas endothelial cell migration is essential for angiogenesis [33]. ZCMP inhibited angiogenesis by suppressing migration of endothelial cells.

### 2.7. ZCMP Enhanced Macrophage Phagocytosis

**Figure 12:**
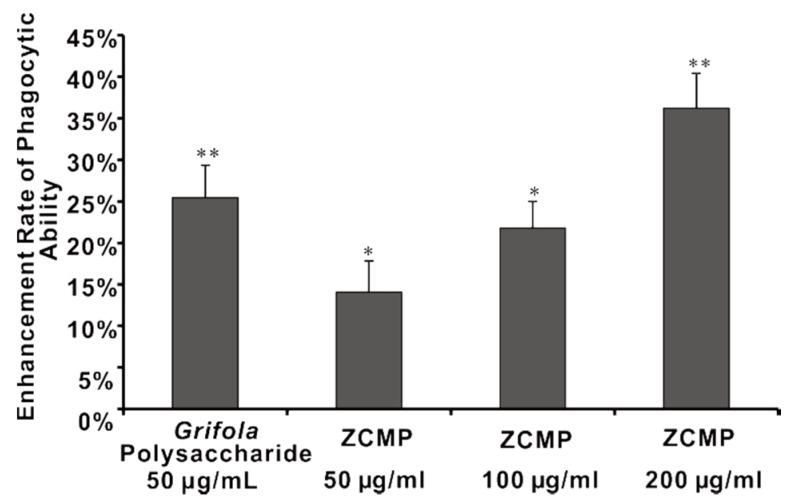
Effects of different doses of ZCMP on the phagocytic ability of the mouse macrophage cell line Raw 264.7. Results are expressed as means ± S.D. * *P* < 0.05; ** *P* < 0.01, as determined by unpaired student’s *t*-test.

The effects of ZCMP treatment on macrophage phagocytosis were examined by using Grifola polysaccharide (50 μg/mL) as positive control (Figure 12). Grifola polysaccharide is a glucan that consists of a backbone of β-1,3 glucosidic bond with β-1,6 side chains, and it has been used clinically for tumor immunotherapy in several countries [34]. The promotion of macrophage phagocytosis was enhanced after increasing the ZCMP dose from 50 μg/mL to 200 μg/mL.

## 3. Experimental Section

### 3.1. Samples and Materials

The seagrass *Z. caespitosa* Miki was collected from Bohai Gulf, China. Monosaccharide standards (Man, Glc, Gal, Xyl, Fuc, GlcA, GalA and Api), sodium borohydride (NaBH_4_), deuterium sodium borohydride (NaBD_4_), 1-ethyl-3-(3-dimethyaminopropyl) carbodimide (EDC), 1-phenyl-3-methyl-5-pyrazolone (PMP), and 5-diphenyl tetrazolium bromide (MTT) were purchased from Sigma-Aldrich (St. Louis, MO, USA). Superdex™ Peptide 10/300 GL column (1.0 × 30 cm) was procured from GE Healthcare (Uppsala, Sweden). Shodex OHpak SB-804 HQ column (8.0 × 300 mm) was obtained from Showa Denko (Tokyo, Japan). Amberlite IR120 resin was purchased from Sigma (St. Louis, MO, USA). All other reagents were of analytical grade.

### 3.2. Extraction, Isolation, and Purification of ZCMP

*Z. caespitosa* Miki was pulverized and passed through a 60 mesh sieve, extracted with 80% ethanol for 3 h at 80 °C (3 times) to remove lipids, and the residue was dried. Approximately 50 g of the residue was pretreated with 75 mL of 0.5% HCl at 50 °C for 3 h to break the cell wall and release the polysaccharides. The residue was washed with water and further extracted with 2% ammonium oxalate solution at 70 °C for 3 h (3 times). Acid polysaccharide was precipitated by adding 3 mol/L HCl until a pH level of 1.0 was attained. The precipitated polysaccharide was dialyzed (7 kD MWCO) against water for 2 days and then freeze-dried.

For purification, the crude polysaccharide (200 mg) was applied to a Q-Sepharose Fast Flow column connected to an ÄKTA-Fast Protein Liquid Chromatography (FPLC) system (General Electric Company, Fairfield, USA), and washed with water, followed by a linear gradient elution with an NaCl solution (from 0 to 4 mol/L) at a flow rate of 120 mL/h. The absorbance of each fraction (10 mL) at 490 nm was tested by using the phenol-sulfuric acid method. The fraction containing polysaccharide was collected, dialyzed, and concentrated. The purified fraction (ZCMP) was dialyzed against distilled water and lyophilized.

### 3.3. General Analysis of ZCMP

Crude protein content was determined by Lowry method [35]. Sulfate content was determined by BaCl_2_-Gelatin method [36]. Purity and relative molecular weight (Mw) were determined by gel filtration chromatography on a Shodex OHpak SB-804 HQ (Showa Denko, Tokyo, Japan) eluted with 0.1 mol/L Na_2_SO_4_ at a flow rate of 0.5 mL/min at 35 °C. The column was calibrated with dextran standards, and the corrected regression equation was log (Mw) = −0.3673*T*_R_ (retention time) + 10.521 (*r* = 0.9918). Monosaccharide composition was determined by a 1-phenyl-3-methyl-5-pyrazolone (PMP)-High Performance Liquid Chromatography (HPLC) method as described by Chen *et al.* [37]. The Fourier Transform Infrared (FTIR) spectra of the polysaccharides ZCMP prepared as a KBr pellet was recorded with a Nicolet Nexus 470 Thermo instrument (Thermo Fisher Scientific, Waltham, MA, USA).

### 3.4. Carboxyl-Group Reductions and Methylation Analysis of ZCMP

ZCMP was first reduced to convert the carboxyl into hydroxyl groups by using the method described by Taylor and Conrad [38]. Methylation of the reduced polysaccharides was performed according to the method of Hakomori [39]. The partially methylated alditol acetates were analyzed by GC-MS equipped with a DB-225MS fused-silica capillary column. Mass spectra of the derivatives were analyzed by using the Complex Carbohydrate Structural Database (CCSD) of the Complex Carbohydrate Research Centre (http://www.ccrc.uga.edu/).

### 3.5. Preparation and Purification of Oligosaccharides of ZCMP

ZCMP (10 mg/mL) was hydrolyzed by adding 0.1 mol/L CH_3_COOH at 100 °C for 2 h, and precipitated with an equivalent volume of ethanol. After centrifugation, the precipitation was named ZCMP-P, and the supernatant was named ZCMP-S. The supernatant was added ethanol to 70% and then precipitated. After centrifugation, the supernatant (ZCMP-SP) and the precipitate (ZCMP-SS) were lyophilized.

ZCMP-P (10 mg/mL) was hydrolyzed by adding 0.2 mol/L HCl at 100 °C for 2 h and precipitated with an equivalent volume of ethanol. After centrifugation, the supernatant (ZCMP-PS) and the precipitation were lyophilized. The precipitate (10 mg/mL) was hydrolyzed by 0.5 mol/L HCl at 100 °C for 3 h, neutralized, and finally lyophilized (ZCMP-PP).

ZCMP (10 mg/mL) was depolymerized with 1% pectinase (w/v) in a buffer solution (0.1 mol/L citric acid: 0.1 mol/L sodium citric acid = 15.5:4.5, v/v, pH 3.5) at 40 °C for 5 h. An equivalent volume of ethanol was added to remove the residual fraction. After centrifugation, the supernatant was lyophilized (ZCMP-E).

ZCMP-S, PS, PP and E were respectively fractionated on a Superdex Peptide column (GE Healthcare, Uppsala, Sweden) and eluted with 0.1 mol/L NH_4_HCO_3_ at a flow rate of 0.1 mL/min, and monitored by a refractive index detector. All fractions were collected and freeze-dried.

### 3.6. Oligosaccharide Reduction

Oligosaccharides reduction was conducted as described by Yu *et al.* [24]. Briefly, 20 μL of a NaBH_4_ reagent (0.05 mol/L NaBH_4_ in 0.01 mol/L NaOH) was added to the oligosaccharide (typically 20 μg). After overnight reduction at 4 °C, CH_3_COOH was added to destroy the borohydride. The mixture was then passed through a mini-column of Amberlite IR120 resin (Sigma, St. Louis, MO, USA), and the boric acid in the eluent was removed by repeated co-evaporation with methyl alcohol.

### 3.7. MS Analysis of Oligosaccharides Derived from ZCMP

ESI-MS of ZCMP oligosaccharides was performed on a LTQ-Orbitrap XL instrument (Thermo Fisher Scientific, Waltham, MA, USA). Samples were dissolved in CH_3_CN/H_2_O (1:1, v/v) at a concentration of 10 pmol/μL and 5 μL was injected. Solvent volatilization temperature and capillary temperatures were 275 °C, and the sheath flow gas flow rate was 8 arb. The flow rate was 8 μL/min in the ESI-MS analysis and 3–5 μL/min in the ESI-CID-MS^2^ analysis. Helium was used as collision gas with a collision energy of 20–25 eV.

### 3.8. NMR Analysis of ZCMP-SS

ZCMP-SS (7 mg) was co-evaporated with D_2_O (99.96%) three times by lyophilization before it was finally dissolved in 500 μL of D_2_O. Acetone was used as internal standard (2.225 ppm for ^1^H-NMR and 30.83 ppm for ^13^C-NMR). The results of the ^1^H-NMR, ^13^C-NMR, DEPT, and HMQC experiments were recorded on JEOL JNM-ECP 600 spectrometer (JEOL, Tokyo, Japan) at 25 °C.

### 3.9. Cell Lines and Culture Conditions

HUVECs and the mouse macrophage cell line Raw 264.7 were purchased from the Cell Bank of the Type Culture Collection Center of the Chinese Academy of Sciences in Shanghai, China. These cells were maintained in DMEM culture medium (Gino Biological Medicine Technology Co., Ltd., Hangzhou, China) supplemented with 10% fetal bovine serum (FBS) (v/v), 100 U/mL penicillin and 100 μg/mL streptomycin, and cultured in an incubator at 37 °C under a humidified atmosphere containing 5% CO_2_.

### 3.10. HUVEC Proliferation (MTT) Assays

Cell proliferation was measured by using the MTT tetrazolium assay. Briefly, HUVECs (cell density: 1 × 10^4^ cells/well) were seeded into 96-well tissue culture plates and cultured with or without ZCMP (50 μg/mL, 100 μg/mL, and 200 μg/mL). After 44 h, the MTT solution was added and the cells were incubated at 37 °C for another 4 h. The insoluble violet formazan product was solubilized by adding 150 μL of DMSO. The color absorbance was recorded at a wavelength 490 nm using a Bio-Tek Elx 808 micro plate reader (BioTek China Shanghai Office, Shanghai, China). The effect of ZCMP on cell viability was calculated in terms of percentage of control, which was arbitrarily assigned a value of 100% viability.

### 3.11. HUVEC Migration Assays

To assess the effect of ZCMP on the mobility of HUVEC cells, a cell migration assay was performed. A total of 1 × 10^5^ HUVEC cells were seeded into each well of 24-well plates and incubated in DMEM medium for 24 h. An artificial line was then created, and the cells were washed and supplied with fresh culture medium and various concentrations of ZCMP (50 μg/mL, 100 μg/mL, and 200 μg/mL). The migration of cells through the line area was examined after 24 h. Images of the migrated cells were captured using a microscope (Olympus, CKX41, Tokyo, Japan).

### 3.12. Macrophage Phagocytosis Assays

Cell suspensions (200 μL), containing 2 × 10^4^ mouse macrophage cells from the cell line Raw 264.7, were added into each well of 96-well plates. After a 6 h incubation to allow the cells to attach to the plate bottom, the cells were cultured with different ZCMP concentrations for another 24 h. Following that, the supernatant was discarded, and 0.075% of a neutral red dye was added to each well (200 μL per well). The plates were incubated for another 30 min. Then, the plates were washed three times with PBS solution (pH 7.2) to remove the redundant neutral red dye. Finally, 200 μL of a lysis solution (acetic acid and ethanol in the ratio of 1:1) was pipetted into each well. The mixtures were thoroughly mixed and evaluated at a wavelength of 540 nm on a Bio-Rad microplate reader (Bio-Rad Laboratories, Hercules, CA, USA).

## 4. Conclusions

The polysaccharide ZCMP extracted from *Z. caespitosa* Miki is composed of AGA and RG-Ι regions. The backbone of the AGA region consists of (1→4)-α-d-GalA residues with an extremely low degree of etherification, whereas the side chains predominantly contained single Api and a few (1→3′)-linked β-d-Api oligosaccharides linked to the O-3 position of GalA. RG-Ι contains a backbone of repeating disaccharide units composed of 4-linked GalA and 2-linked Rha, with minor 5-linked α-l-Ara residues, and 4-linked β-d-Gal residues were attached as side chains. ZCMP showed anti-angiogenesis activity by inhibiting migration of HUVECs, and immunoregulation activity by enhancing the phagocytosis of macrophages. Further research studies on the inhibition of tumor metastasis of ZCMP and its structure-activity relationship are warranted.

## Supplementary Files

Supplementary File 1
