# Supplementary material for: Extraction, Isolation, Structural Characterization and Anti-Tumor Properties of an Apigalacturonan-Rich Polysaccharide from the Sea Grass Zostera caespitosa Miki
Source: Mar Drugs. 2015 Jun 11;13(6):3710–31. doi: 10.3390/md13063710 (PMC4483652; doi:10.3390/md13063710)
Supplement: Supplementary File 1 [file marinedrugs-13-03710-s001.pdf]

# Supplementary Information

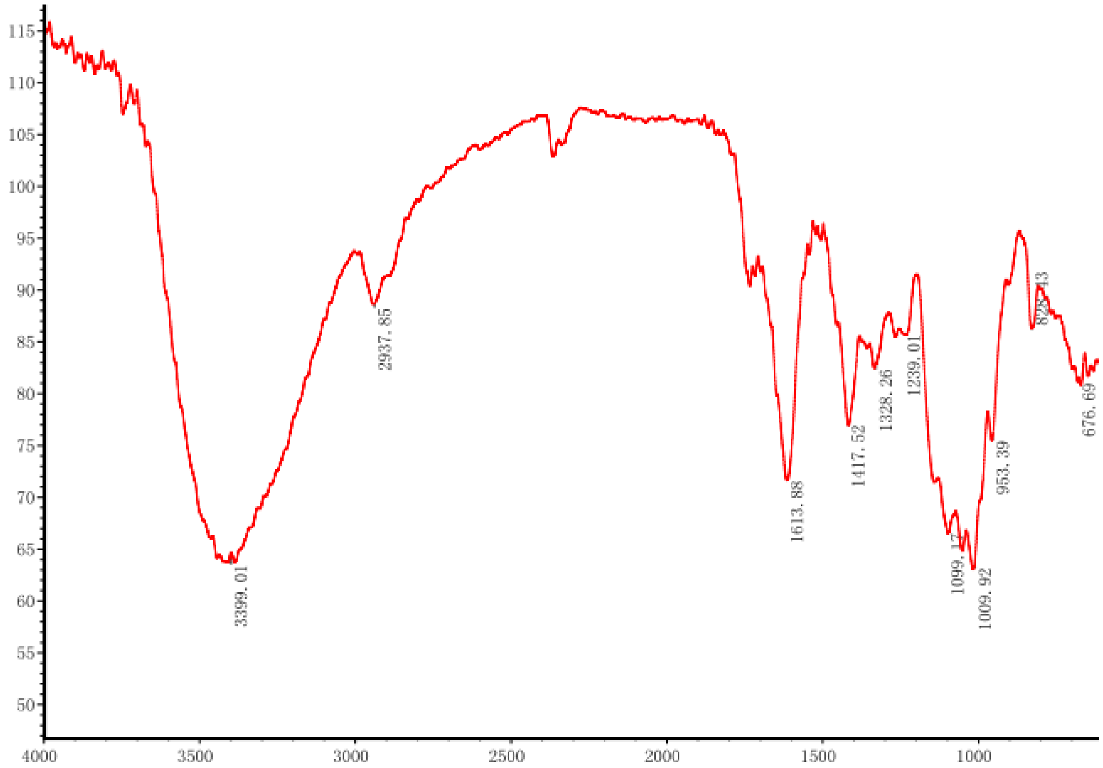


**Figure S1.** Infrared absorption spectrum of ZCMP.

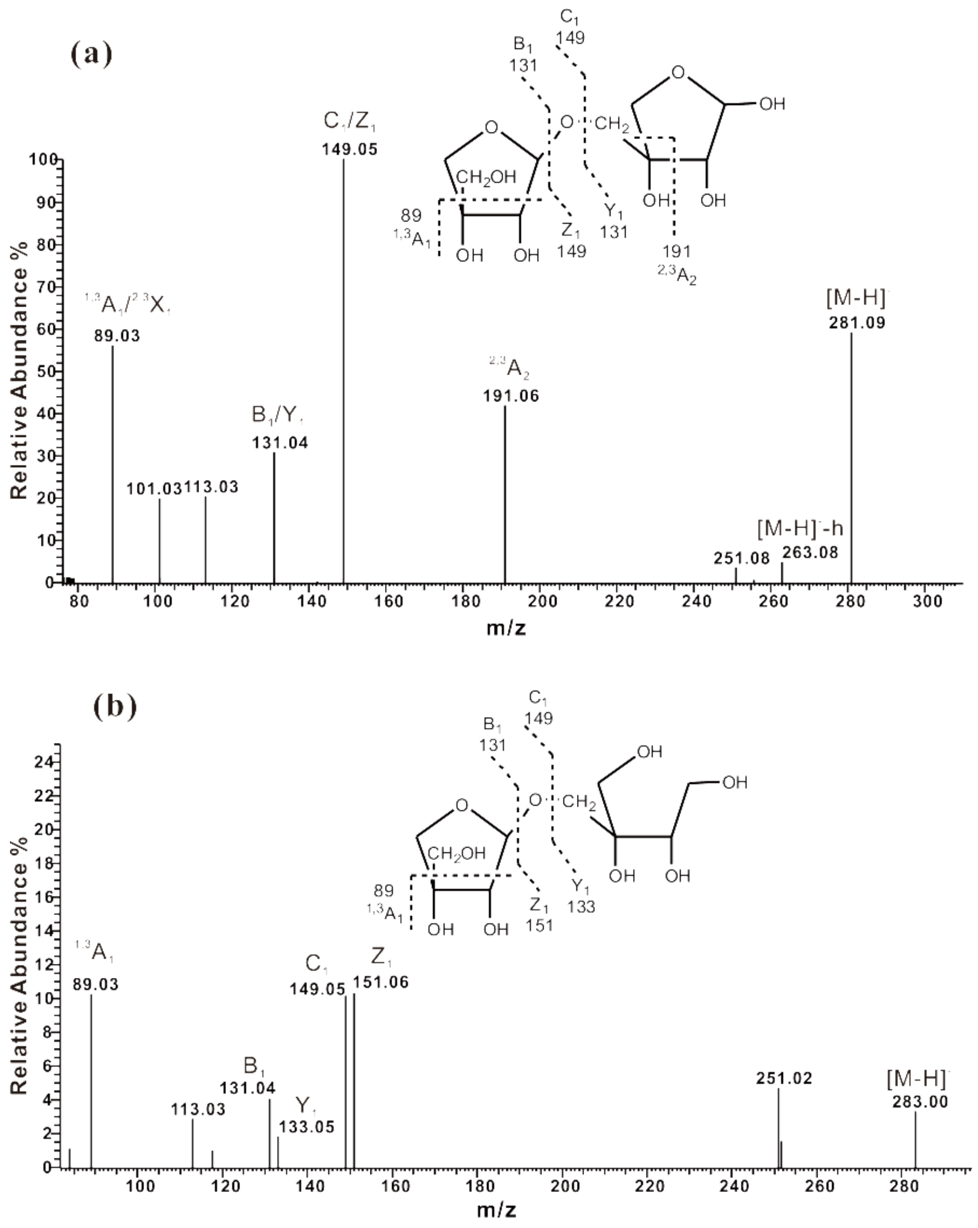


**Figure S2.** Negative-ion ESI-CID-MS$^2$ product-ion spectra of disaccharide Api$_2$ from ZCMP-S. (**a**) Sequence analysis of disaccharide Api$_2$ at *m/z* 281; (**b**) Sequence analysis of disaccharide alditol at *m/z* 283.

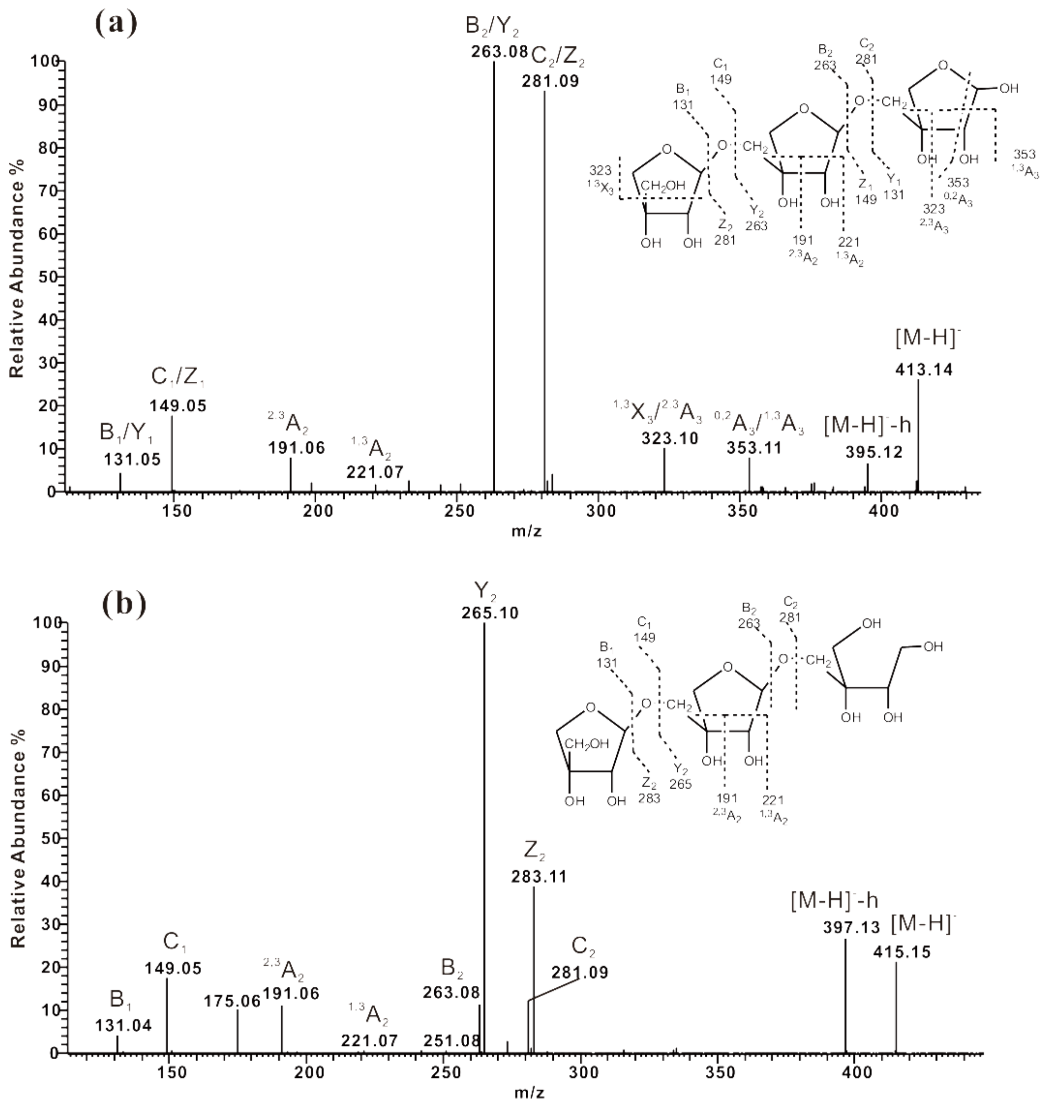


**Figure S3.** Negative-ion ESI-CID-MS$^2$ product-ion spectra of trisaccharide Api$_3$ from ZCMP-S. (**a**) Sequence analysis of trisaccharide Api$_3$ at *m/z* 413; (**b**) Sequence analysis of trisaccharide alditol at *m/z* 415.

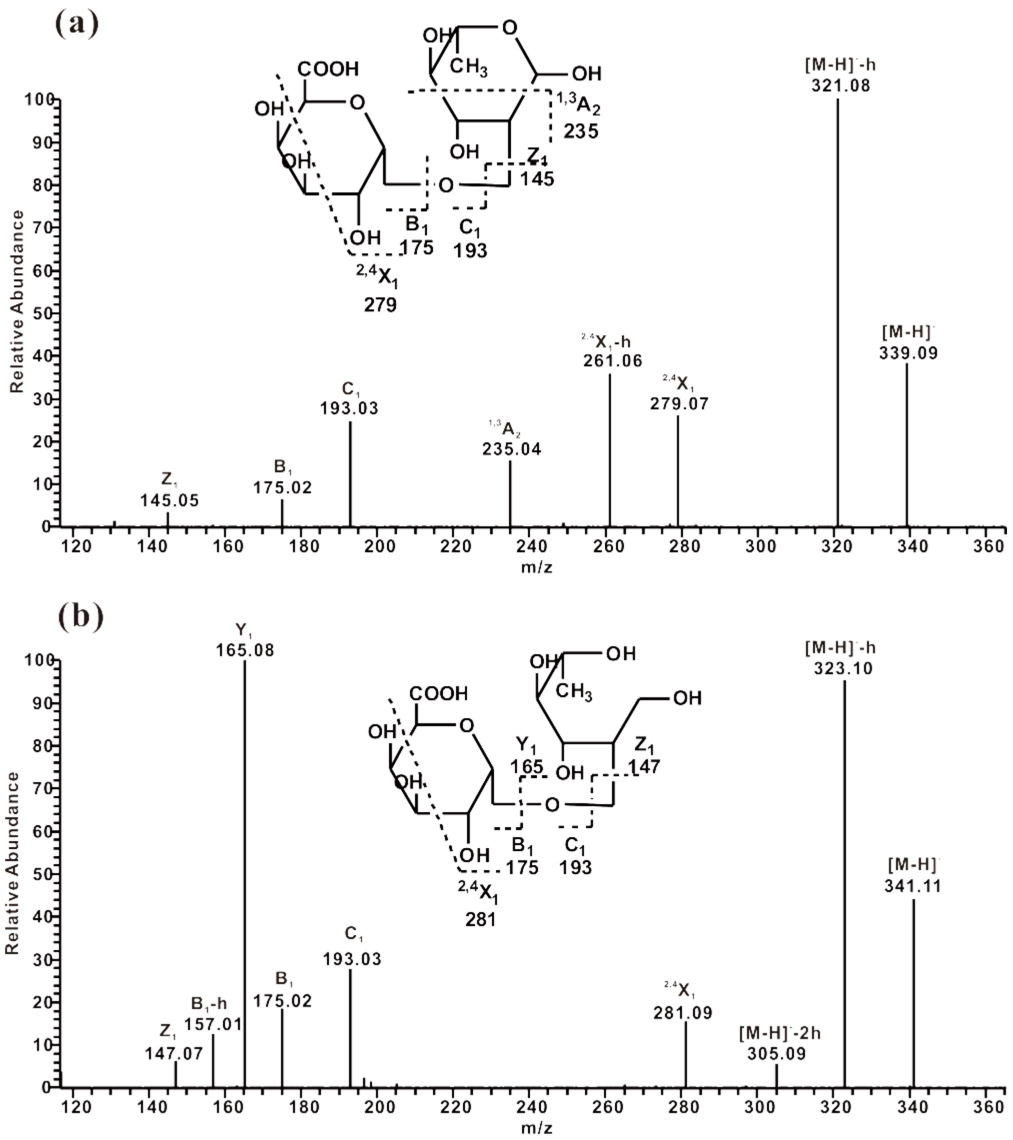


**Figure S4.** Negative-ion ESI-CID-MS$^2$ product-ion spectra of disaccharide GalA-Rha from ZCMP-PS. (**a**) Sequence analysis of disaccharide GalA-Rha at *m/z* 339; (**b**) Sequence analysis of disaccharide alditol at *m/z* 341.

© 2015 by the authors; licensee MDPI, Basel, Switzerland. This article is an open access article distributed under the terms and conditions of the Creative Commons Attribution license (http://creativecommons.org/licenses/by/4.0/).
